# The truncated splice variants, *NT*-*PGC*-*1α* and *PGC*-*1α4*, increase with both endurance and resistance exercise in human skeletal muscle

**DOI:** 10.1002/phy2.140

**Published:** 2013-11-05

**Authors:** Mia Ydfors, Helene Fischer, Henrik Mascher, Eva Blomstrand, Jessica Norrbom, Thomas Gustafsson

**Affiliations:** 1 Department of Physiology and Pharmacology, Karolinska InstitutetStockholm, Sweden; 2 Department of Laboratory Medicine, Karolinska InstitutetStockholm, Sweden; 3 The Swedish School of Sport and Health SciencesStockholm, Sweden

**Keywords:** Exercise, human, PGC-1*α*, skeletal muscle

## Abstract

Recently, a truncated peroxisome proliferator-activated receptor gamma coactivator-1 alpha (PGC-1*α*) splice variant, PGC-1*α*4, that originates from the alternative promoter was shown to be induced by resistance exercise and to elicit muscle hypertrophy without coactivation of “classical” PGC-1*α* targets involved in mitochondrial biogenesis and angiogenesis. In order to test if distinct physiological adaptations are characterized by divergent induction of PGC-1*α* splice variants, we investigated the expression of truncated and nontruncated *PGC-1α* splice variants and *PGC-1α* transcripts originating from the alternative and the proximal promoter, in human skeletal muscle in response to endurance and resistance exercise. Both total *PGC-1α* and truncated *PGC-1α* mRNA expression were increased 2 h after endurance (*P* < 0.01) and resistance exercise (*P* < 0.01), with greater increases after endurance exercise (*P* < 0.05). Expression of nontruncated *PGC-1α* increased significantly in both exercise groups (*P* < 0.01 for both groups) without any significant differences between the groups. Both endurance and resistance exercise induced truncated as well as nontruncated *PGC-1α* transcripts from both the alternative and the proximal promoter. Further challenging the hypothesis that induction of distinct *PGC-1α* splice variants controls exercise adaptation, both nontruncated and truncated *PGC-1α* transcripts were induced in AICAR-treated human myotubes (*P* < 0.05). Thus, contrary to our hypothesis, resistance exercise did not specifically induce the truncated forms of *PGC-1α*. Induction of truncated *PGC-1α* splice variants does not appear to underlie distinct adaptations to resistance versus endurance exercise. Further studies on the existence of numerous splice variants originating from different promoters are needed.

## Introduction

In the last decade, technological advancements have yielded new tools (e.g., tissue knockout models, microarray, and bioinformatics), creating the possibility to identify and characterize regulatory factors controlling muscle plasticity. One such emerging factor has been the transcriptional coactivator peroxisome proliferator-activated receptor gamma coactivator-1 alpha (PGC-1*α*). PGC-1*α* was initially recognized as a master regulator of mitochondrial biogenesis (Puigserver et al. 1998; Wu et al. 1999) because of its binding to, and coactivation of, nuclear receptors and transcription factors such as the peroxisome proliferator-activated receptors and the nuclear respiratory factors (Wu et al. 1999; Vega et al. 2000; Lin et al. 2005). The apparent importance of PGC-1*α* in exercise-induced mitochondrial biogenesis is supported by reports showing that exercise induces *PGC-1α* expression in human, rat, and mouse skeletal muscle (Baar et al. 2002; Irrcher et al. 2003; Pilegaard et al. 2003; Short et al. 2003; Norrbom et al. 2004, 2011; Akimoto et al. 2005) and further demonstrated in conditional knockout models (Handschin et al. 2007).

Via coactivation of transcription factors, for example, myocyte enhancer factor 2 and estrogen-related receptor, PGC-1*α* also plays an important role in other adaptive processes, such as vascular growth and induction of a “slower” muscle fiber phenotype (Lin et al. 2005; Arany et al. 2008). Such adaptations are typically associated with endurance-type exercise but other types of physical activity (e.g., sprint, and resistance exercise) also result in an increase in the levels of the *PGC-1α* transcript (Gibala 2009; Lundberg et al. 2013). Moreover, PGC-1*α* was recently shown to participate in the regulation of skeletal muscle hypertrophy (Ruas et al. 2012), which suggests a more complex regulation of exercise-induced transcription of *PGC-1α* in the control of adaptation.

The existence of different *PGC-1α* splice variants has been reported in skeletal muscle, but the nomenclature is rather confusing (Baar et al. 2002; Chinsomboon et al. 2009; Yoshioka et al. 2009; Norrbom et al. 2011). In mice, three splice variants (*PGC-1α-a, -b,* and *-c*) have been identified (Miura et al. 2008) where at least two of these are related to activation of the alternative promoter and induced with exercise. Another variant, NT-PGC-1*α*, is produced via alternative 3′ splicing, resulting in a shorter, truncated, protein compared with the full-length PGC-1*α* protein (Zhang et al. 2009). Additionally, four different *PGC-1α* splice variants were recently characterized by Ruas et al. (2012); *PGC-1α1, α2, α3,* and *α4*. *PGC-1α4* is a truncated splice variant with the alternative 3′ splicing as earlier demonstrated for NT-PGC-1. Ruas et al. (2012) demonstrated that this splice variant controls muscle hypertrophy without coactivation of known PGC-1*α* targets involved in mitochondrial biogenesis or angiogenesis. *PGC-1α4* originates from the alternative promoter (exon 1b), whereas NT-PGC1-*α* originates from the proximal promoter (exon 1a) (Zhang et al. 2009). Importantly, the truncated splice variants result in shorter proteins influencing which specific transcription factors that are co-activated.

In our laboratory, we recently demonstrated that exercise-induced transcription from both the alternative promoter and the proximal promoter occur in human skeletal muscle (Norrbom et al. 2011). The alternative promoter seems to be less dependent on AMPK activation (Miura et al. 2008; Norrbom et al. 2011) compared to the proximal promoter, but also more strongly influenced by catecholamines. All together, a plausible hypothesis is therefore that various types of exercise induce the expression of *PGC-1α* splice variants with differences in biological effects (Zhang et al. 2009; Chang et al. 2010; Ruas et al. 2012).

Our aim in this study was to investigate the expression of truncated and nontruncated *PGC-1α* splice variants in human skeletal muscle, and whether they originate from the alternative (exon 1b) and/or the proximal promoter (exon 1a). Also, we aimed to elucidate whether endurance and resistance types of exercise stimulate the expression of these forms differently. Our hypothesis was that *PGC-1α* increases with both types of exercise and that the truncated splice variants, and transcripts originating from exon 1b, would be induced to a greater extent by resistance exercise in contrast to endurance exercise.

## Methods

### Ethical approval

The Regional Ethical Review Board in Stockholm, Sweden approved the study. The subjects gave written informed consent before participating in the respective experimental setup. The study conformed to the standards set by the Declaration of Helsinki.

### Subjects and exercise protocols

Two different experimental setups were included in the study: endurance and resistance exercise. A total of 16 healthy male subjects, who were not actively engaged in bodybuilding or any specific/focused training (e.g., cycling or resistance exercise) were included in the study. Eight subjects participated in endurance exercise, their mean (±SD) age was 27 (±3) years, mean height was 179 (±12) cm, mean weight was 79 (±12) kg, mean body mass index was 24.7 (±2.4) kg/m^2^, and the mean maximal oxygen uptake (VO_2max_) was 3.91 (±1.02) L/min. Another eight subjects took part in resistance exercise, their mean (±SD) age was 23 (±3) years, mean height was 181 (±4) cm, mean weight was 75 (±11) kg, mean body mass index was 22.9 (±3.1) kg/m^2^, and the mean VO_2max_ was 3.91 (±0.42) L/min.

Endurance exercise: eight subjects exercised for 1 h on a cycle ergometer at 70% of their VO_2max_. Prior to the bout of exercise, the subjects participated in a preparatory test in which VO_2max_ was determined on a cycle ergometer using an online system (SensorMedics V_max_, Encore, 229; Viasys Respiratory Care, Yorba Linda, CA). The VO_2max_ test was performed at least 5 days before the experimental exercise bout. The subjects were asked to refrain from vigorous physical activity for 2 days before the study.

Resistance exercise: eight subjects performed one bout of resistance exercise, 4 × 10 repetitions in a leg press machine at 80% of their one-repetition maximum (1RM) with a 5-min rest period between sets. To minimize the risk of injury, the subjects performed light exercise for 10 min (cycling at 100 W) to warm-up before the leg press exercise. Prior to the actual experiment, the subjects participated in two preparatory tests. The first test was designed to determine their 1RM, whereas during the second preparatory test, the subjects performed the exercise routine scheduled to be performed during the study. The second test was performed at least 5 days before the actual exercise bout. In addition, the subjects’ VO_2max_ was determined during treadmill running using an online system (Amis 2001 Automated Metabolic Cart; Innovision A/S, Odense, Denmark). The subjects were asked to refrain from vigorous physical activity for 2 days before the study. The subjects performing resistance exercise also took part in one of our earlier investigations (Mascher et al. 2008).

### Skeletal muscle biopsies

Endurance exercise skeletal muscle biopsies were obtained from the *m. vastus lateralis* using a percutaneous needle biopsy technique (Bergström et al. 1967). Biopsies were taken at rest before exercise and 2 h after the exercise bout. Resistance exercise muscle biopsies were obtained from the *m. vastus lateralis* using a Weil-Blakesley conchotome (AB Wisex, Mölndal, Sweden), as described earlier (Henriksson 1979), at rest before and 2 h after the exercise bout. Biopsies were taken from the same leg (right leg in four subjects and left leg in four subjects) with the first biopsy ∼11–14 cm above mid-patella. Two hours following exercise, the biopsy was taken from a new incision proximal to the first one. Tissue samples from both groups were immediately frozen in liquid nitrogen and then stored at −80°C until further analysis.

### Cell culture

Parts of the muscle biopsies (40 mg) obtained at rest were stored in sterile phosphate-buffered saline containing 1% penicillin–streptomycin at 4°C overnight. Extraction of cells from the biopsy samples was performed as described previously (Norrbom et al. 2011), with some modifications. In brief, the samples were washed, minced, and dissociated enzymatically in 5 mL of 0.25% trypsin and 1 mmol/L ethylenediaminetetraacetic acid (all cell media were from Invitrogen, Stockholm, Sweden) at 37°C with 5% CO_2_ and gentle agitation for 20 min. Undigested tissue was allowed to settle for 5 min, and the supernatant was collected in growth medium (Dulbecco's modified Eagle's medium [DMEM-F-12] containing 1% penicillin/streptomycin and 20% fetal calf serum [FCS]). Digestion of the slurry was repeated twice. The cells were cultured in T75 flasks (Sarstedt, Stockholm, Sweden), and growth medium was changed every third or fourth day until 60% confluency was reached. For the experiment, myoblasts were cultured in growth medium to 80% confluency. Subsequently, the medium was replaced with differentiation medium (DMEM-F-12 containing 1% penicillin/streptomycin and 2% FCS). On day 5 of culture with differentiation medium, the cells were treated with 5-amino-imidazole-4-carboxamide-1-*β*-D-ribofuranoside (AICAR; 1 mmol/L) or no treatment (control) for 24 h.

### RNA extraction and mRNA quantification

**Figure 1:**
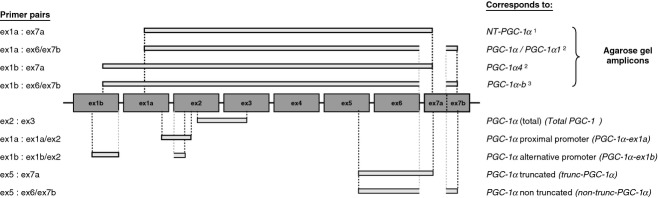
Schematic representation of exons 1–7 of the human *PGC-1α* gene. Primer pairs are depicted to the left, and the resulting splice variants measured and promoter sites are depicted to the right. Upper panel: resulting amplicons with RT-PCR. Lower panel: amplicons measured with real-time RT-PCR, SYBR® Green. Exon 1b is transcribed from the alternative promoter, and exon 1a is transcribed from the proximal promoter. Exon 7a (ex7a) is the exon insert resulting in the truncated forms of *PGC-1α* (*trunc*-*PGC*-*1α*) and exon 7b (ex7b) is present in nontruncated *PGC-1α* (*non-trunc-PGC*-*1α*). The corresponding names of previously described splice variants are stated to the right in the upper panel. ^1^Zhang et al. (2009), ^2^Ruas et al. (2012), ^3^Miura et al. (2008).

RNA was extracted from freeze-dried muscle biopsy samples and cultured cells using a standard TRIzol protocol (Sigma, Stockholm, Sweden). The RNA was quantified by measuring absorbance at 260 nm using NanoDrop 2000 (Thermo Scientific, Göteborg, Sweden) and RNA integrity was assessed after electrophoresis on an agarose gel and visualized in an ultraviolet transilluminator. Two micrograms of total RNA was reverse transcribed using Superscript reverse transcriptase using random hexamer primers (Life Technologies, Stockholm, Sweden) in a total volume of 20 *μ*L. Primers were designed to identify *PGC-1α* splice variants transcribed from the proximal promoter (exon 1a; here called *PGC-1α-ex1a*) and from the upstream-located alternative promoter (exon 1b; here called *PGC-1α-ex1b*) and to cover exon–exon boundaries (synthesized by Cybergene, Stockholm, Sweden). Primers for the truncated forms of *PGC-1α* were designed to cover the insert of exon 7a (here called *trunc-PGC-1α*) and primers for the nontruncated forms of *PGC-1α* covered the exon 6 and 7b boundaries, excluding the insert sequence (here called *non-trunc-PGC-1α*). The primers (with the exception of the primer for exon 7a) were modified from the mouse primers published previously (Miura et al. 2008, Zhang et al. 2009) using the NCBI genome database, to correspond to the human genome sequence. Thus, the *trunc-PGC-1α* primers measure the expression of both *PGC-1α4,* as described by Ruas et al. (2012), and *NT-PGC-1α*, as described earlier (Zhang et al. 2009). The locations of all primers are shown in Figure 1.

**Figure 2:**
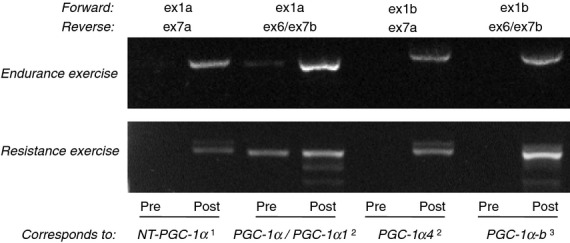
Agarose gel with RT-PCR products of truncated and nontruncated splice variants of *PGC-1α* transcribed from two promoters. Forward primers located in exon 1a (ex1a) and exon 1b (ex1b) were combined with reverse primers for truncated (ex7a) and nontruncated (ex6/ex7b) *PGC-1α* splice variants. The bands correspond to the calculated amplicon sizes, showing that both truncated and nontruncated forms are expressed from both the proximal and the alternative promoter. The corresponding names of previously described splice variants are stated below the gel. ^1^Zhang et al. (2009), ^2^Ruas et al. (2012), ^3^Miura et al. (2008).

Reverse transcriptase polymerase chain reaction (RT-PCR) products, from samples taken before and 2 h after endurance and resistance exercise, using primers detecting the inserted exon 7a in combination with primers located in exon 1a and exon 1b, respectively, were run on an agarose gel (Fig. 2). Exon 1a is transcribed from the proximal promoter and captures the *NT-PGC-1α* isoform, whereas exon 1b is transcribed from the alternative promoter and makes up the initial part of the *PGC-1α4* isoform. A similar approach was used to detect nontruncated splice variants, using a reversed primer covering the exon 6 and 7b boundaries. The RT-PCR reaction volume was 25 *μ*L, including 5 *μ*L of sample cDNA diluted 1:100, forward and reverse primers (final concentration, 0.4 *μ*mol/L), 250 nmol/L of dNTPs, and 2.5 U of AmpliTaq Gold (Applied Biosystems, Foster City, CA). After the RT-PCR products were run on an agarose gel, the bands were excised and the RT-PCR products isolated and verified by sequencing (KIGene, Karolinska University Hospital, Stockholm, Sweden). Primers used in the RT-PCR are shown in Figure 1.

Real-time RT-PCR was used for mRNA quantification. The reaction volume was 15 *μ*L, including 4 *μ*L of sample cDNA diluted 1:100, forward primer (final concentration, 0.4 *μ*mol/L), reverse primer (final concentration, 0.4 *μ*mol/L), and SYBR Green PCR Master Mix (Applied Biosystems, Stockholm, Sweden). All quantification reactions were verified with a melting curve and glyceraldehyde 3-phosphate dehydrogenase (*GAPDH*) was used as an endogenous control. Primer efficiency was tested by calculating the ΔC_T_ of housekeeping and target genes at different concentrations. The *k* value was less than 0.1 when ΔC_T_ was plotted against log(concentration) for all primer pairs used in PCR analyses.

### Statistical analyses

The statistical analyses were conducted on normalized values; a ΔC_T_ value was obtained by subtracting the *GAPDH* C_T_ value from the corresponding target gene C_T_ value. For expression comparisons, *PGC-1α-ex1a* was subtracted from *PGC-1α-ex1b* and *non-trunc-PGC-1α* was subtracted from *trunc-PGC-1α*. The expression of each target was then determined using 2^−ΔCT^, which provides the level of expression of the target gene relative to the level of expression of the endogenous control in each sample. A mixed model two-way analysis of variance (ANOVA) was used to evaluate the mRNA response to exercise (pre-exercise and 2 h post exercise) in the two conditions (endurance and resistance), and for expression of nontruncated and truncated splice variants in cell culture (control and AICAR stimulation). Planned comparison (i.e., post hoc test) was used to identify significant interactions, or, when no interaction was found, to identify differences corresponding to significant main effects in the ANOVA model described above. Differences were considered significant at *P* < 0.05. Results are presented as mean ± SEM.

## Results

### PGC-1α mRNA expression from the proximal and the alternative promoter after endurance and resistance exercise

Bands corresponding to the calculated amplicon sizes for the truncated splice variants (*NT-PGC-1α* and *PGC-1α4*) were identified on an agarose gel. Moreover, amplicons representing the nontruncated forms expressed from both promoters were detected (Fig. 2). From the gel it was evident that truncated and nontruncated transcripts were expressed from both the proximal and the alternative promoter. Sequencing of the PCR products verified that the correct sequences were amplified.

### Expression of PGC-1α splice variants after endurance and resistance exercise

**Figure 3:**
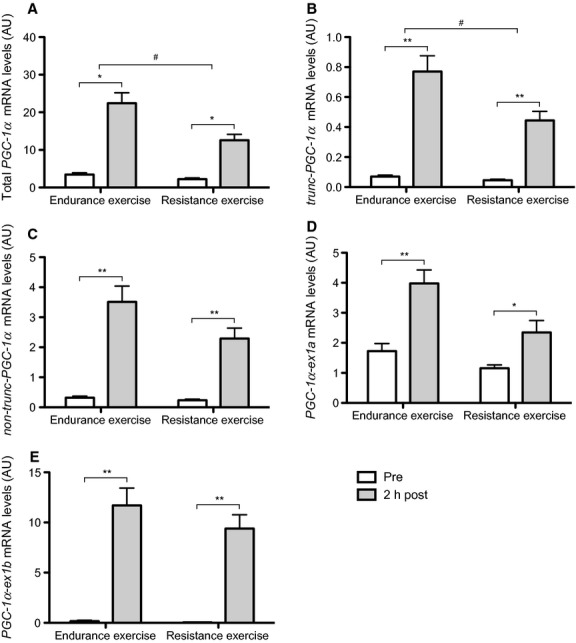
mRNA expression of *PGC-1α* splice variants in human skeletal muscle in response to an acute bout of endurance and resistance exercise. mRNA expression of (A) Total *PGC-1α*, (B) *trunc*-*PGC*-*1α*, (C) *non-trunc*-*PGC*-*1α*, (D) *PGC*-*1α*-*ex1a*, and (E) *PGC*-*1α*-*ex1b* before (pre) and 2 h after (2 h post) a single bout of endurance and resistance exercise. Graphical data represent arbitrary units (AU) normalized to *GAPDH* mRNA expression. Values are expressed as mean ± SEM for eight subjects. *Represents *P* < 0.05 pre versus 2 h post. **Represents *P* < 0.01 pre versus 2 h post. ^#^Represents *P* < 0.05 endurance versus resistance exercise; the ANOVA revealed a significant interaction between time and exercise condition.

**Figure 4:**
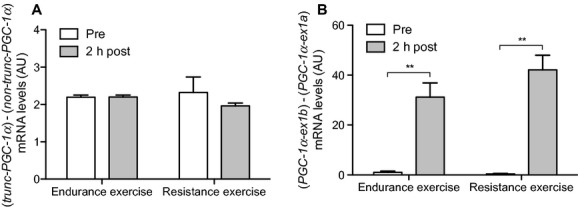
Comparison of mRNA expression from the alternative and the proximal promoter and the truncated and the nontruncated splice variants of *PGC-1α* before and after a single bout of endurance and resistance exercise. Comparison of the mRNA expression (A) of the truncated to the nontruncated splice variants (*trunc*-*PGC*-*1α* – *non*-*trunc*-*PGC*-*1α*) and (B) from the alternative and the proximal promoter (*PGC*-*1α*-*ex1b* – *PGC*-*1α*-*ex1a*) before (pre) and 2 h after (2 h post) a single bout of endurance and resistance exercise. Graphical data represent arbitrary units (AU). Values are expressed as mean ± SEM for eight subjects. *Represents *P* < 0.05 pre versus 2 h post.

Total *PGC-1α* expression (primers located in exons 2 and 3) was increased significantly after both 60 min of endurance cycling exercise and a single bout of resistance exercise (*P* < 0.01 for both groups). The increase was significantly higher in the endurance exercise group than in the resistance exercise group (*P* < 0.05; Fig. 3A). Expression of *trunc-PGC-1α* (exon 7a) was increased in response to both endurance and resistance exercise (*P* < 0.01 for both groups), with a greater increase observed after endurance exercise (*P* < 0.05; Fig. 3B). The *non-trunc-PGC-1α* levels (exons 5/7b) were increased significantly in both exercise groups (*P* < 0.01 for both groups; Fig. 3C) without any significant differences between the groups. The comparison of the truncated and nontruncated transcripts (*trunc-PGC-1α – non-trunc-PGC-1α*) before and after exercise showed no significant differences, either within or between the two exercise groups (Fig. 4A).

Expression from exon 1a (*PGC-1α-ex1a*) was increased significantly in response to both endurance exercise (*P* < 0.01) and resistance exercise (*P* < 0.05; Fig. 3D), without any significant differences between the groups. Expression from exon 1b (*PGC-1α-ex1b*) was virtually undetectable at rest in both exercise groups. However, 2 h after the exercise bout, the levels had increased in both the endurance and resistance exercise group (*P* < 0.01 for both groups; Fig. 3E) without any significant differences between the groups. Comparison of the expression from the alternative and the proximal promoter (*PGC-1α-ex1b – PGC-1α-ex1a*) before and after a single bout of either endurance or resistance exercise revealed evident distribution changes between transcripts originating from exons 1b and 1a; the *PGC-1α-ex1b* expression increase was much greater than the *PGC-1α-ex1a* increase after exercise (*P* < 0.01 for both groups; Fig. 4B).

### Cell culture experiments

**Figure 5:**
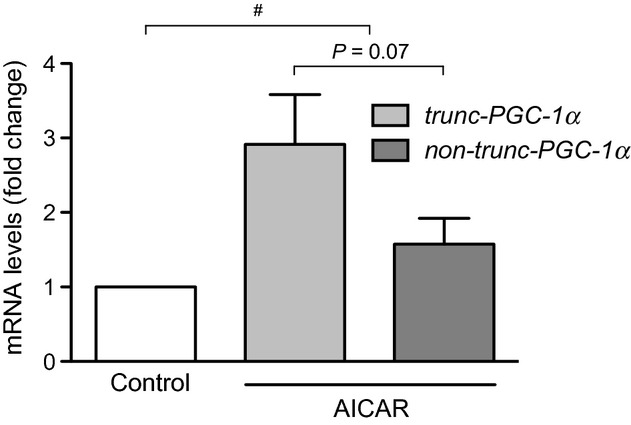
mRNA expression of *PGC-1α* splice variants in AICAR-stimulated human myotubes. mRNA expression in human myotubes of *trunc*-*PGC*-*1α* and *non-trunc*-*PGC*-*1α* after stimulation with AICAR for 24 h compared to unstimulated control myotubes. Graphical data represent arbitrary units (AU) normalized to *GAPDH* mRNA expression and are presented as fold change. *n* = 4. #Represents interaction between expression of *trunc*-*PGC*-*1α* and *non-trunc*-*PGC*-*1α* in AICAR-stimulated myotubes, *P* < 0.05.

Cultured human myotubes were treated with AICAR as a metabolic stimulus (AMPK stimulator) for 24 h. This treatment induced a significantly higher expression of truncated and nontruncated PGC-1*α* splice variants (*P* < 0.05). There was a tendency toward a greater increase for the truncated splice variants compared with the nontruncated splice variants (*P* = 0.07; Fig. 5).

## Discussion

In the current study, we investigated the expression of truncated and nontruncated splice variants of *PGC-1α* in human skeletal muscle, as well as in response to two different types of exercise, endurance and resistance. We further wanted to study if the splice variants originated from the alternative promoter (exon 1b) and/or from the proximal promoter (exon 1a). We showed that both truncated and nontruncated variants of *PGC-1α* were expressed in resting human skeletal muscle and that they were transcribed from both the proximal and the alternative promoter. Furthermore, the expression of the truncated and nontruncated splice variants increased with both exercise types, with a greater increase observed following endurance exercise. Exercise-induced expression from exon 1a and exon 1b did not differ between endurance and resistance exercise.

Several PGC-1*α* splice variants have been reported (Miura et al. 2008; Zhang et al. 2009; Ruas et al. 2012). We demonstrate expression of both truncated and nontruncated splice variants in resting human skeletal muscle. The expression of *trunc-PGC-1α* and *non-trunc-PGC-1α* are important given that differences in protein domains influence which transcription factors that are coactivated (Zhang et al. 2009; Ruas et al. 2012). This may represent one of the mechanisms underlying the manner in which specific splice variants of PGC-1*α* induce distinct skeletal muscle-remodeling processes (Wu et al. 1999; Lin et al. 2005; Ruas et al. 2012). In a recent study, the truncated splice variant, *PGC-1α4,* was shown to increase only after resistance training, with no changes observed after endurance training (Ruas et al. 2012). In this study, the expression of truncated and nontruncated splice variants, as well as total *PGC-1α,* increased 2 h after a single bout of either endurance or resistance exercise. In fact, a greater increase in truncated splice variants was observed following endurance exercise.

Confounding factors always exist when comparing different types of exercise modes, which is why we wanted to more specifically elaborate on whether a metabolic pathway was able to influence the expression of *trunc-PGC-1α*. AICAR is a well-known stimulator of AMPK in skeletal muscle (Merrill et al. 1997) which has evolved as the main metabolic regulator of PGC-1*α*. Therefore, cultured human myotubes were stimulated with AICAR and both *trunc-PGC-1α* and *non-trunc-PGC-1α* mRNA expression was demonstrated to increase compared with control.

In addition to *PGC-1α4*, another truncated form of PGC-1*α*, *NT-PGC-1α*, has been demonstrated in animal models and in contrast to *PGC-1α4* it has been shown to coactivate more “classic” PGC-1*α* targets involved in, for example, mitochondrial biogenesis (Zhang et al. 2009). These truncated splice variants (*NT-PGC-1α* and *PGC-1α4*) have been proposed to have different start sequences, that is, transcribed from different promoters: 1a (proximal promoter) and 1b (alternative promoter). However, they do share exon 7a, which inserts a stop codon in the mRNA sequence (Zhang et al. 2009; Ruas et al. 2012). The RT-PCR primers used in the current study, as well as in earlier reports (Zhang et al. 2009; Ruas et al. 2012), are not able to differentiate between the proximal and the alternative promoter as they are positioned further downstream. Importantly, in the current study, we clearly demonstrate with the agarose gel that the truncated splice variants originate from both the proximal (exon 1a) and the alternative promoter (exon 1b).

An additional aim in this study was to test whether different stimuli activate the two characterized *PGC-1α* promoter regions differently. Earlier, we and others have reported that exercise-induced transcripts originating from the proximal promoter are more dependent on metabolic stimuli, for example, AMPK (Miura et al. 2007, 2008; Norrbom et al. 2011). We therefore hypothesized a greater increase in transcripts originating from the alternative promoter after resistance exercise than after endurance exercise. However, this was not supported in this study. Thus, a specific increase in transcripts originating from exon 1b with resistance training could not be detected, which adds to our findings that truncated and nontruncated splice variants are activated with both resistance and endurance exercise.

The focus of the current study was to measure the truncated and nontruncated splice variants, rather than to specifically evaluate all of the reported splice variants in the literature. Nonetheless, it has been argued that the nontruncated splice variants, for example, *PGC-1α1*, originate exclusively from exon 1a. However, in this study, we identified a nontruncated splice variant (which was controlled by sequencing) that included exon 1b and had a similar size to that of *PGC-1α1*. Still, it remains unknown which transcripts that translate into functional protein(s).

It could be argued that the differences between the present study and the study by Ruas et al., regarding effects of resistance exercise, are related to the number of exercise bouts and/or the time point at which the biopsy was sampled. In our study, sampling was done 2 h after a single bout of exercise, whereas in the previous study sampling was made 48 h after the last bout of an 8-week training program. In humans, the temporal pattern of *PGC-1α* expression in response to exercise is well characterized and shown to return to baseline levels within 24–48 h after exercise (Pilegaard et al. 2003; Short et al. 2003; Russell et al. 2005; Kuhl et al. 2006; Perry et al. 2010). Based on these reports, the degree of changes observed in *PGC-1α4* levels is, to some extent, surprising. However, it is not possible to exclude that the kinetics of *PGC-1α4* mRNA, or of other truncated splice variants, are different compared with the nontruncated splice variants. Nevertheless, this would not explain the discrepancy regarding an exercise type-specific (e.g., resistance exercise) activation of truncated splice variants.

In conclusion, both truncated and nontruncated splice variants of *PGC-1α* were expressed in human skeletal muscle, and originated from both the alternative and the proximal promoter. However, in contrast to our hypothesis, we could detect an increase in truncated and nontruncated splice variants with both resistance and endurance exercise. All together, induction of truncated PGC-1*α* splice variants does not appear to underlie distinct adaptations to resistance versus endurance exercise. Further studies on the existence of numerous splice variants originating from different promoters are needed.
